# Reducing the exome search space for Mendelian diseases using genetic linkage analysis of exome genotypes

**DOI:** 10.1186/gb-2011-12-9-r85

**Published:** 2011-09-14

**Authors:** Katherine R Smith, Catherine J Bromhead, Michael S Hildebrand, A Eliot Shearer, Paul J Lockhart, Hossein Najmabadi, Richard J Leventer, George McGillivray, David J Amor, Richard J Smith, Melanie Bahlo

**Affiliations:** 1 Bioinformatics Division, The Walter and Eliza Hall Institute of Medical Research, 1G Royal Parade, Parkville, Victoria 3052, Australia; 2 Department of Otolaryngology-Head and Neck Surgery, University of Iowa, Iowa City, Iowa 52242, USA; 3 Department of Molecular Physiology and Biophysics, University of Iowa Carver College of Medicine, Iowa City, IA 52242, USA; 4 Murdoch Childrens Research Institute, Royal Children's Hospital, Parkville, Victoria 3052, Australia; 5 Bruce Lefroy Centre for Genetic Health Research, Murdoch Childrens Research Institute, Royal Children's Hospital, Parkville, Victoria 3052, Australia; 6 Genetics Research Center, University of Social Welfare and Rehabilitation Sciences, Tehran 19834, Iran; 7 Department of Paediatrics, University of Melbourne, Royal Children's Hospital, Parkville, Victoria 3052, Australia; 8 Children's Neuroscience Centre, Royal Children's Hospital, Parkville, Victoria 3052, Australia; 9 Interdepartmental PhD Program in Genetics, University of Iowa, Iowa City, Iowa 52242, USA; 10 Department of Mathematics and Statistics, The University of Melbourne, Parkville, Victoria 3010, Australia

## Abstract

Many exome sequencing studies of Mendelian disorders fail to optimally exploit family information. Classical genetic linkage analysis is an effective method for eliminating a large fraction of the candidate causal variants discovered, even in small families that lack a unique linkage peak. We demonstrate that accurate genetic linkage mapping can be performed using SNP genotypes extracted from exome data, removing the need for separate array-based genotyping. We provide software to facilitate such analyses.

## Background

Whole exome sequencing (WES) has recently become a popular strategy for discovering potential causal variants in individuals with inherited Mendelian disorders, providing a cost- effective, fast-track approach to variant discovery. However, a typical human genome differs from the reference genome at over 10,000 potentially functional sites [1]; identifying the disease-causing mutation among this plethora of variants can be a significant challenge. For this reason, exome sequencing is often preceded by genetic linkage analysis, which allows variants outside of linkage peaks to be excluded. The linkage peaks delineate tracts of identity by descent sharing that match the proposed genetic model. This combination strategy has been successfully used to identify variants causing autosomal dominant [2-4] and recessive [5-11] diseases, as well as those affecting quantitative traits [12-14]. Linkage analysis has also been used in conjunction with whole genome sequencing (WGS) [15].

Other WES studies have not performed formal linkage analysis, but have nonetheless considered inheritance information, such as searching for large regions of homozygosity shared by affected family members using genotypes obtained from genotyping arrays [16-18] or exome data [19,20]. This method does not incorporate genetic map or allele frequency information, which could help to eliminate regions from consideration, and is applicable only to recessive diseases resulting from consanguinity. Recently, it has been suggested that identity by descent regions be identified from exome data using a non-homogeneous hidden Markov model (HMM), allowing variants outside these regions to be eliminated [21,22]. This method incorporates genetic map information but not allele frequency information and requires a strict genetic model (recessive and fully penetrant) and sampling scheme (exomes of two or more affected siblings must be sequenced). It would be suboptimal for use with diseases resulting from consanguinity, for which filtering by homozygosity by descent would be more effective than filtering by identity by descent. Finally, several WES studies have been published that make no use of inheritance information whatsoever, despite the fact that DNA from other informative family members was available [23-31].

Classical linkage analysis using the multipoint Lander-Green algorithm [32], which is a HMM, incorporates genetic map and allele frequency information and allows for great flexibility in the disease model. Unlike the methods just mentioned, linkage analysis allows dominant, recessive or X-linked inheritance models, as well as permitting variable penetrances, non-parametric analysis and formal haplotype inference. There are few constraints upon the sampling design, with unaffected individuals able to contribute information to parametric linkage analyses. The Lander-Green algorithm has produced many important linkage results, which have facilitated the identification of the underlying disease-causing mutations.

We investigated whether linkage analysis using the Lander-Green algorithm could be performed using genotypes inferred from WES data, removing the need for the array-based genotyping step [33]. We inferred genotypes at the location of HapMap Phase II SNPs, [34] as this resource provides comprehensive annotation, including the population allele frequencies and genetic map positions required for linkage analysis. We adapted our existing software [35] to extract HapMap Phase II SNP genotypes from WES data and format them for linkage analysis.

We anticipated two potential disadvantages to this approach. Firstly, exome capture only targets exonic SNPs, resulting in gaps in marker coverage outside of exons. Secondly, genotypes obtained using massively parallel sequencing (MPS) technologies such as WES tend to have a higher error rate than those obtained from genotyping arrays [36]. The use of erroneous genotypes in linkage analyses may reduce power to detect linkage peaks or result in false positive linkage peaks [37].

**Figure 1:**
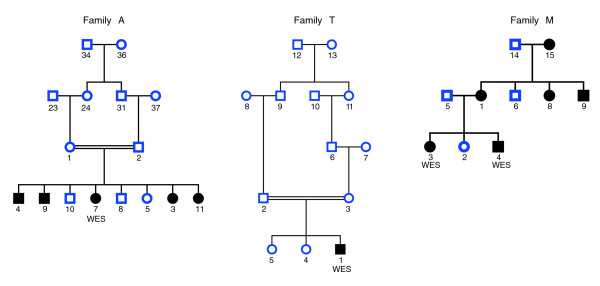
**Partial pedigrees for families A, T and M**.

We compared the results of linkage analysis using array-based and exome genotypes for three families with different neurological disorders showing Mendelian inheritance (Figure 1). We sequenced the exomes of two affected siblings from family M, an Anglo-Saxon ancestry family showing autosomal dominant inheritance. The exome of a single affected individual, the offspring of first cousins, from Iranian family A was sequenced, as was the exome of a single affected individual, the offspring of parents thought to be first cousins once removed, from the Pakistani family T. Families A and T showed recessive inheritance. Due to the consanguinity present in these families, we can perform linkage analysis using genotypes from a single affected individual, a method known as homozygosity mapping [33].

## Results and discussion

### Exome sequencing coverage of HapMap Phase II SNPs

Allele frequencies and genetic map positions were available for 3,269,163 HapMap Phase II SNPs that could be translated to UCSC hg19 physical coordinates. The Illumina TruSeq platform used for exome capture targeted 61,647 of these SNPs (1.89%). After discarding indels and SNPs whose alleles did not match the HapMap annotations, a median 56,931 (92.3%) of targeted SNPs were covered by at least five high-quality reads (Table 1). A median of 64,065 untargeted HapMap Phase II SNPs were covered by at least five reads; a median 78% of these untargeted SNPs were found to lie within 200 bp of a targeted feature, comprising a median 57% of all untargeted HapMap SNPs within 200 bp of a targeted feature.

**Table 1:** Number of HapMap Phase II SNPs covered ≥ 5 by distance to targeted base

Distance to	Number of SNPs (%)				HapMap
		
targeted base	M-3	M-4	A-7	T-1	Phase II (N)
0 bp	56,648 (91.9)	56,835 (92.2)	57,027 (92.5)	58,142 (94.3)	61,647
1 to 200 bp	50,077 (56.7)	50,805 (57.5)	46,144 (52.2)	57,923 (65.6)	88,349
> 200 bp	13,683 (0.4)	13,565 (0.4)	13,987 (0.4)	17,007 (0.5)	3,119,167
Total	120,408 (3.7)	121,205 (3.7)	117,158 (3.6)	133,072 (4.1)	3,269,163

The denominator for percentages is the total number of HapMap Phase II SNPs in that distance category.

In total, we obtained a minimum of 117,158 and a maximum of 133,072 SNP genotypes from the four exomes. The array-based genotyping interrogated 598,821 genotypes for A-7 and T-1 (Illumina Infinium HumanHap610W-Quad BeadChip) and 731,306 genotypes for M-3 and M-4 (Illumina OmniExpress BeadChip). Table 2 compares the inter-marker distances between exome genotypes for each sample to those for the genotyping array. The exome genotypes have much more variable inter-marker distances than the genotyping arrays, with a smaller median value.

**Table 2:** Intermarker distances for the two genotyping arrays and for exome genotypes covered ≥ 5

	Median	1st quartile	3rd quartile
Illumina OmniExpress	2,233	814	5,125
Illumina 610	2,744	1,019	6,027
M-3	1,853	236	11,390
M-4	1,830	235	11,260
A-7	1,943	240	12,000
T-1	1,647	227	10,210

Intermarker distances are in base pairs.

### Optimization of genotype concordance

We inferred genotypes at the positions of SNPs located on the genotyping array used for each individual so that we could investigate genotype concordance between the two technologies. We found that ambiguous (A/T or C/G SNPs) comprised a high proportion of SNPs with discordant genotypes, despite being a small proportion of SNPs overall. For example, for A-7 at coverage ≥ 5 and *t *= 0.5 (see below), 77% (346 of 450) of discordant SNPs were ambiguous SNPs, while ambiguous SNPs composed just 2.7% of all SNPs (820 of 30,279). Such SNPs are prone to strand annotation errors, as the two alleles are the same on both strands of the SNP. We therefore discarded ambiguous SNPs, which left 29,459 to 52,892 SNPs available for comparison (Table 3).

**Table 3:** Increasing the prior heterozygous probability modestly improves concordance between exome and array genotypes

*t*	M-3 (N = 52,617)	M-4 (N = 52,892)	A-7 (N = 29,459)	T-1 (N = 32,763)
0.00001	0.9737	0.9734	0.9698	0.9741
0.001 (default)	0.9882	0.9874	0.9865	0.9885
0.01	0.9927	0.9926	0.9918	0.9925
0.05	0.9951	0.9950	0.9942	0.9945
0.1	0.9958	0.9958	0.9950	0.9952
0.2	0.9968	0.9965	0.9958	0.9961
0.3	0.9971	0.9968	0.9961	0.9964
0.4	0.9973	0.9971	0.9964	0.9968
0.5	0.9974	0.9973	0.9965	0.9969

Proportion of SNPs where WES and genotyping array genotypes are concordant for the four exomes, for varying values of *t *(prior probability of a heterozygous genotype). Conditional on coverage with ≥ 5 reads.

Several popular genotype-calling algorithms for MPS data require the prior probability of a heterozygous genotype to be specified [38,39]. We investigated the effect of varying this parameter, *t*, upon concordance of genotyping array and WES genotypes (given WES coverage ≥ 5; Table 3). Increasing this value from the default 0.001 results in a modest improvement in the percentage of WES genotypes being correctly classified, with most of the improvement occurring between *t *= 0.001 and *t *= 0.05. The highest concordance is achieved at *t *= 0.5, where all four samples achieve 99.7% concordance, compared to 98.7 to 98.9% concordance at the default *t *= 0.001.

We note that *t *= 0.5 may not be optimal for calling SNP genotypes on haploid chromosomes. At *t *= 0.5, the male M-4 had five × chromosome genotypes erroneously called as heterozygous out of 1,026 (0.49%), while the male T-1 had one such call out of 635 genotypes (0.16%). The same SNPs were not called as heterozygous by the genotyping arrays. No heterozygous × chromosome calls were observed at the default value of *t *= 0.001.

### Linkage analysis and LOD score concordance

Prior to performing linkage analysis on exome and array SNP genotypes, we selected one SNP per 0.3 cM to ensure linkage equilibrium while retaining a set of SNPs dense enough to effectively infer inheritance. The resulting subsets of WES genotypes (Table 4) contained 8,016 to 8,402 SNPs with average heterozygosities of 0.40 or 0.41 among the CEPH HapMap genotypes, obtained from Utah residents with ancestry from northern and western Europe (CEU). The resulting subsets of array genotypes (Table 4) contained more SNPs (12,173 to 12,243), with higher average heterozygosities (0.48 or 0.49).

**Table 4:** Number and average heterozygosity of array and WES SNPs selected for linkage analysis

	M-3 and M-4		A-7		T-1	
	
	WES	Array	WES	Array	WES	Array
SNPs available	114,681	677,144	117,158	593,638	133,071	587,680
SNPs selected	8,016	12,173	8,135	12,243	8,402	12,194
Average heterozygosity	0.40	0.49	0.40	0.48	0.41	0.48

Average heterozygosity refers to the HapMap CEU population and not to the individual being studied. For M-3 and M-4, 'SNPs available' is the number of SNPs covered ≥ 5 in both individuals.

**Figure 2:**
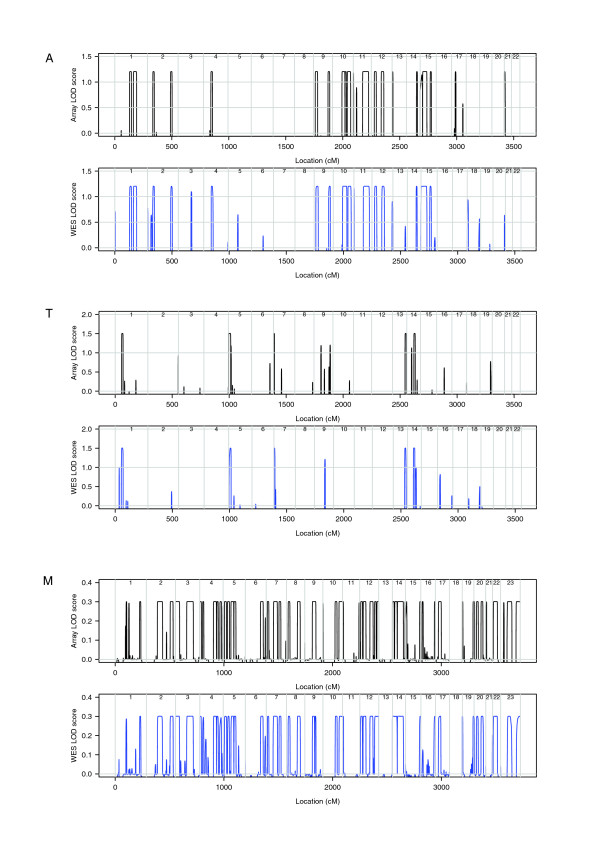
**Genome-wide comparison of LOD scores using array-based and WES-derived genotypes for families A, T and M**.

Despite this difference, there was good agreement between LOD scores achieved at linkage peaks using the different sets of genotypes (Figure 2, Table 5). The median difference between the WES and array LOD scores across positions where either achieved the maximum score was close to zero for all three families (range -0.0003 to -0.002). The differences had a 95% empirical interval of (-0.572,0.092) for family A, with the other two families achieving narrower intervals (Table 5).

**Table 5:** Distribution of LOD score differences (WES - array) at linkage peaks

Family	Median	2.5th centile	97.5th centile
A	-0.0005	-0.572	0.092
T	-0.002	-0.390	0.035
M	-0.0003	-0.117	0.0034

Summary of differences at analysis positions where either the WES or the array LOD scores reach their genome-wide maximum.

### Efficacy of filtering identified variants by location of linkage peaks

If our genetic model is correct, then variants lying outside of linkage peaks cannot be the causal mutation and can be discarded, thus reducing the number of candidate disease-causing variants. Table 6 lists the number of nonsynonymous exonic variants (single nucleotide variants or indels) identified in each exome, as well as the number lying with linkage peaks identified using WES genotypes. The percentage of variants eliminated depends upon the power of the pedigree being studied: 81.2% of variants are eliminated for the dominant family M, which is not very powerful; 94.5% of variants are eliminated for the recessive, consanguineous family A; while 99.43% of variants are eliminated for the more distantly consanguineous, recessive family T. Hence, linkage analysis substantially reduces the fraction of variants identified that are candidates for the disease-causing variant of interest.

**Table 6:** Efficacy of variant elimination due to linkage peak filtering

Family	Model	Consanguinity	Number of linkage peaks	Max LOD	Number of not synonymous exonic variants	Number of (%) not synonymous exonic variants in linkage regions
A	Recessive	First cousin offspring	15	1.2	10,982	604 (5.50)
T	Recessive	First cousins once removed offspring	5	1.51	11,353	65 (0.57)
M	Dominant	None	41	0.3	13,186	2,478 (18.79)

## Conclusions

Linkage analysis is of great potential benefit to WES studies that aim to discover genetic variants resulting in Mendelian disorders. As variants outside of linkage peaks can be eliminated, it reduces the number of identified variants that need to be investigated further. Linkage analysis of WES genotypes provides information regarding the location of the disease locus to be extracted from WES data even if the causal variant is not captured, suggesting regions of interest that may be targeted in follow-up studies. However, many such studies are being published that employ less sophisticated substitutes for linkage analysis or do not consider inheritance information at all. Anecdotal evidence suggests that a substantial proportion of MPS studies of individuals with Mendelian disorders fail to identify a causal variant, though an exact number is not known due to publication bias.

We describe how to extract HapMap Phase II SNP genotypes from massively parallel sequencing data, providing software to facilitate this process and generate files ready to be analyzed by popular linkage programs. Our method allows linkage analysis to be performed without requiring genotyping arrays. The flexibility of linkage analysis means that our method can be applied to any disease model and a variety of sampling schemes, unlike existing methods of considering inheritance information for WES data. Linkage analysis incorporates population allele frequencies and genetic map positions, which allows superior identification of statistically unusual sharing of haplotypes between affected individuals in a family.

We demonstrate linkage using WES genotypes for three small nuclear families - a dominant family from which two exomes were sequenced and two consanguineous families from which a single exome was sequenced. As these families are not very powerful for linkage analysis, multiple linkage peaks with relatively low LOD scores were identified. Nonetheless, discarding variants outside of the linkage peaks eliminated between 81.2% and 99.43% of all nonsynonymous exonic variants detected in these families. The number of variants remaining could be reduced further by applying standard strategies, such as discarding known SNPs with minor allele frequencies above a certain threshold. Our work demonstrates the value of considering inheritance information, even in very small families that may consist, at the extreme, of a single inbred individual. As the price of exome sequencing falls, it will become feasible to sequence more individuals from each family, resulting in fewer linkage peaks with higher LOD scores.

Exome capture using current technologies yields large numbers of useful SNPs for linkage mapping. Over half of all SNPs covered by five or more reads were not targeted by the exome capture platform. Approximately 78% of these captured untargeted SNPs lay within 200 bp of a targeted feature. This reflects the fact that fragment lengths typically exceed probe lengths, resulting in flanking sequences at both ends of a probe or bait being captured and sequenced. The serendipitous result is that a substantial number of non-exonic SNPs become available, which can and should be used for linkage analysis.

We found that setting the prior probability of heterozygosity to 0.5 during genotype inference resulted in the best concordance between WES and array genotypes. The authors of the MAQ SNP model recommend using *t *= 0.2 for inferring genotypes at known SNPs [38], while the default value used to detect variants is *t *= 0.001. Our results highlight the need to tailor this parameter to the specific application, either genotyping or rare variant detection. Although we anticipated WES genotypes being less accurate than array genotypes, all four samples achieved a high concordance of 99.7% for SNPs covered by five or more reads at *t *= 0.5

We found that LOD scores obtained from WES genotypes agreed well with those obtained from array genotypes from the same individual(s) at the location of linkage peaks, with the median difference in LOD score zero to two or three decimal places for all three families. This was despite the fact that the array-based genotype sets used for analysis contained more markers and had higher average heterozygosities than the corresponding WES genotype sets, reflecting the fact that genotyping arrays are designed to interrogate SNPs with relatively high minor allele frequencies that are relatively evenly spaced throughout the genome. By contrast, genotypes extracted from WES data tend to be clustered around exons, resulting in fewer and less heterozygous markers after pruning to achieve linkage equilibrium. We conclude that if available, array-based genotypes from a high resolution SNP array are preferable to WES genotypes; but if not, linkage analysis of WES genotypes produces acceptable results.

Once WGS is more economical, we will be able to perform linkage analysis using genotypes extracted from WGS data, which will obviate the problem of gaps in SNP coverage outside of exons. The software tools we provide can accommodate WGS genotypes without requiring modification. In the future, initiatives such as the 1000 Genomes Project [1] may provide population-specific allele frequencies for SNPs not currently included in HapMap, further increasing the number of SNPs available for analyses, as well as the number of populations studied.

The classic Lander-Green algorithm requires markers to be in linkage equilibrium [40]. Modeling linkage disequilibrium would allow incorporation of all markers without the need to select a subset of markers in linkage equilibrium. This would allow linkage mapping using distant relationships, such as distantly inbred individuals who would share a sub-linkage (< 1 cM) tract of DNA homozygous by descent. Methods that incorporate linkage disequilibrium have already been proposed, including a variable length HMM that can be applied to detect distantly related individuals [41]. Further work is being targeted towards approximations of distant relationships to connect sets of related pedigrees [42]. These methods will extract the maximum information from MPS data from individuals with inherited diseases.

We have integrated the relatively new field of MPS in families with classical linkage analysis. Where feasible, we strongly advocate the use of linkage mapping in combination with MPS studies that aim to discover variants causing Mendelian disorders. This approach does not require purpose-built HMMs, but can utilize existing software implementations of the Lander-Green algorithm. Where genotyping array genotypes are not available, we recommend utilizing MPS data to their full capacity by using MPS genotypes to perform linkage analysis. This will reduce the number of candidate disease-causing variants that need to be evaluated further. Should the causal variant not be identified by a WES study, linkage analysis will highlight regions of the genome where targeted resequencing is most likely to identify this variant.

## Materials and methods

### Informed consent, DNA extraction and array-based genotyping

Written informed consent was provided by the four participants or their parents. Ethics approval was provided by the Royal Children's Hospital Research Ethics Committee (HREC reference number 28097) in Melbourne. Genomic DNA was extracted from participants' blood samples using the Nucleon™ BACC Genomic DNA Extraction Kit (GE Healthcare, Little Chalfont, Buckinghamshire, England).

All four individuals were genotyped using Illumina Infinium HumanHap610W-Quad BeadChip (A-7, T-1) or OmniExpress (M-3, M-4) genotyping arrays (fee for service, Australian Genome Research Facility, Melbourne, Victoria, Australia). These arrays interrogate 598,821 and 731,306 SNPs respectively, with 342,956 markers in common. Genotype calls were generated using version 6.3.0 of the GenCall algorithm implemented in Illumina BeadStudio. A GenCall score cutoff (no-call threshold) of 0.15 was used.

### Exome capture, sequencing and alignment

Target DNA for the four individuals was captured using Illumina TruSeq, which is designed to capture a target region of 62,085,286 bp (2.00% of the genome), and sequenced using an Illumina HiSeq machine (fee for service, Axeq Technologies, Rockville, MD, United States). Individual T-1 was sequenced using one-quarter of a flow cell lane while the other three individuals were sequenced using one-eighth of a lane. Paired-end reads of 110 bp were generated.

Reads were aligned to UCSC hg19 using Novoalign version 2.07.05 [43]. Quality score recalibration was performed during alignment, and reads that aligned to multiple locations were discarded. Following alignment, presumed PCR duplicates were removed using MarkDuplicates.jar from Picard [44]. Table S1 in Additional file 1 shows the number of reads at each stage of processing, while Tables S2 and S3 in the same file show coverage statistics for the four exomes.

### WES genotype inference and linkage analysis

SNP genotypes were inferred from WES data using the samtools mpileup and bcftools view commands from release 916 of the SAMtools package [45], which infers genotypes using a revised version of the MAQ SNP model [38]. We required base quality and mapping quality ≥ 13. SAMtools produces a variant call format (VCF) file, from which we extracted genotypes using a Perl script.

These genotypes were formatted for linkage analysis using a modified version of the Perl script linkdatagen.pl [35] with an annotation file prepared for HapMap Phase II SNPs. This script chose one SNP per 0.3 cM to be used for analysis, with SNPs selected to maximize heterozygosity according to CEU HapMap genotypes [34]. Array-based genotypes were prepared for linkage analysis in the same way, using annotation files for the appropriate array.

The two Perl scripts used to extract genotypes from VCF files and format them for linkage analysis are freely available on our website [46], as is the annotation file for HapMap Phase II SNPs. Users may also download VCF files containing WES SNP genotypes for the four individuals described here (both for HapMap Phase II and genotyping array SNPs), as well as files containing genotyping array genotypes for comparison.

Multipoint parametric linkage analysis using WES and array genotypes was performed using MERLIN [47]. A population disease allele frequency of 0.00001 was specified, along with a fully penetrant recessive (family A, family T) or dominant (family M) genetic model. LOD scores were estimated at positions spaced 0.3 cM apart, and CEU allele frequencies were used.

### WES variant detection

SAMtools mpileup/bcftools was also used to detect variants from the reference sequence with the default setting of *t *= 0.001. Variants were annotated by ANNOVAR [48] using the UCSC Known Gene annotation. For the purposes of filtering variants, linkage peaks were defined as the intervals in which the genome-wide maximum LOD score was obtained, plus 0.3 cM on either side.

## Abbreviations

bp: base pair; HMM: hidden Markov model; MPS: massively parallel sequencing; SNP: single nucleotide polymorphism; VCF: variant call format; WES: whole exome sequencing; WGS: whole genome sequencing.

## Authors' contributions

KRS conceived of the study and performed all analyses described in the article. MB provided guidance and ideas. CJB wrote software tools. MSH, AES, and RJHS performed whole exome sequencing. MSH performed array-based SNP genotyping. RJHS, RJL, HN, GM and DJA collected families and clinical data. PJL contributed reagents and materials. KRS and MB drafted the initial article. All authors discussed the results and commented on the manuscript.

## Supplementary Material

Additional file 1 **Supplementary tables**. Click here for file

## References

[B1] Durbin RM Abecasis GR Altshuler DL Auton A Brooks LD Gibbs RA Hurles ME McVean GA 1000 Genomes Project Consortium A map of human genome variation from population-scale sequencing. Nature 2010 467 1061 1073 10.1038/nature09534 20981092 PMC3042601

[B2] Johnson JO Mandrioli J Benatar M Abramzon Y Van Deerlin VM Trojanowski JQ Gibbs JR Brunetti M Gronka S Wuu J Ding J McCluskey L Martinez-Lage M Falcone D Hernandez DG Arepalli S Chong S Schymick JC Rothstein J Landi F Wang Y-D Calvo A Mora G Sabatelli M Monsurrò Rosaria Maria Battistini S Salvi F Spataro R Sola P Borghero G Exome Sequencing Reveals VCP Mutations as a Cause of Familial ALS. Neuron 2010 68 857 864 10.1016/j.neuron.2010.11.036 21145000 PMC3032425

[B3] Wang JL Yang X Xia K Hu ZM Weng L Jin X Jiang H Zhang P Shen L Feng Guo J Li N Li YR Lei LF Zhou J Du J Zhou YF Pan Q Wang J Wang J Li RQ Tang BS TGM6 identified as a novel causative gene of spinocerebellar ataxias using exome sequencing. Brain 2010 133 3510 3518 10.1093/brain/awq323 21106500

[B4] Southgate L Machado RD Snape KM Primeau M Dafou D Ruddy DM Branney PA Fisher M Lee GJ Simpson MA He Y Bradshaw TY Blaumeiser B Winship WS Reardon W Maher ER FitzPatrick DR Wuyts W Zenker M Lamarche-Vane N Trembath RC Gain-of-Function Mutations of ARHGAP31, a Cdc42/Rac1 GTPase Regulator, Cause Syndromic Cutis Aplasia and Limb Anomalies. The American Journal of Human Genetics 2011 88 574 585 10.1016/j.ajhg.2011.04.013 PMC3146732 21565291

[B5] Bilguvar K Ozturk AK Louvi A Kwan KY Choi M Tatli B Yalnizoglu D Tuysuz B Caglayan AO Gokben S Kaymakcalan H Barak T Bakircioglu M Yasuno K Ho W Sanders S Zhu Y Yilmaz S Dincer A Johnson MH Bronen RA Kocer N Per H Mane S Pamir MN Yalcinkaya C Kumandas S Topcu M Ozmen M Sestan N Whole-exome sequencing identifies recessive WDR62 mutations in severe brain malformations. Nature 2010 467 207 210 10.1038/nature09327 20729831 PMC3129007

[B6] Bolze A Byun M McDonald D Morgan NV Abhyankar A Premkumar L Puel A Bacon CM Rieux-Laucat F Pang K Britland A Abel L Cant A Maher ER Riedl SJ Hambleton S Casanova J-L Whole-Exome-Sequencing-Based Discovery of Human FADD Deficiency. Am J Hum Genet 2010 87 873 881 10.1016/j.ajhg.2010.10.028 21109225 PMC2997374

[B7] Kalay E Yigit G Aslan Y Brown KE Pohl E Bicknell LS Kayserili H Li Y Tuysuz B Nurnberg G Kiess W Koegl M Baessmann I Buruk K Toraman B Kayipmaz S Kul S Ikbal M Turner DJ Taylor MS Aerts J Scott C Milstein K Dollfus H Wieczorek D Brunner HG Hurles M Jackson AP Rauch A Nurnberg P CEP152 is a genome maintenance protein disrupted in Seckel syndrome. Nat Genet 2011 43 23 26 10.1038/ng.725 21131973 PMC3430850

[B8] Otto EA Hurd TW Airik R Chaki M Zhou W Stoetzel C Patil SB Levy S Ghosh AK Murga-Zamalloa CA van Reeuwijk J Letteboer SJF Sang L Giles RH Liu Q Coene KLM Estrada-Cuzcano A Collin RWJ McLaughlin HM Held S Kasanuki JM Ramaswami G Conte J Lopez I Washburn J Macdonald J Hu J Yamashita Y Maher ER Guay-Woodford LM Candidate exome capture identifies mutation of SDCCAG8 as the cause of a retinal-renal ciliopathy. Nat Genet 2010 42 840 850 10.1038/ng.662 20835237 PMC2947620

[B9] Walsh T Shahin H Elkan-Miller T Lee MK Thornton AM Roeb W Abu Rayyan A Loulus S Avraham KB King M-C Kanaan M Whole exome sequencing and homozygosity mapping identify mutation in the cell polarity protein GPSM2 as the cause of nonsyndromic hearing loss DFNB82. Am J Hum Genet 2010 87 90 94 10.1016/j.ajhg.2010.05.010 20602914 PMC2896776

[B10] Abou Jamra R Philippe O Raas-Rothschild A Eck SH Graf E Buchert R Borck G Ekici A Brockschmidt FF Nöthen MM Munnich A Strom TM Reis A Colleaux L Adaptor Protein Complex 4 Deficiency Causes Severe Autosomal-Recessive Intellectual Disability, Progressive Spastic Paraplegia, Shy Character, and Short Stature. The American Journal of Human Genetics 2011 88 788 795 10.1016/j.ajhg.2011.04.019 PMC3113253 21620353

[B11] Sirmaci A Walsh T Akay H Spiliopoulos M Şakalar YB Hasanefendioğlu-Bayrak A Duman D Farooq A King M-C Tekin M MASP1 mutations in patients with facial, umbilical, coccygeal, and auditory findings of Carnevale, Malpuech, OSA, and Michels syndromes. Am J Hum Genet 2010 87 679 686 10.1016/j.ajhg.2010.09.018 21035106 PMC2978960

[B12] Bowden DW An SS Palmer ND Brown WM Norris JM Haffner SM Hawkins GA Guo X Rotter JI Chen YDI Wagenknecht LE Langefeld CD Molecular basis of a linkage peak: exome sequencing and family-based analysis identify a rare genetic variant in the ADIPOQ gene in the IRAS Family Study. Hum Mol Genet 2010 19 4112 4120 10.1093/hmg/ddq327 20688759 PMC2947405

[B13] Musunuru K Pirruccello JP Do R Peloso GM Guiducci C Sougnez C Garimella KV Fisher S Abreu J Barry AJ Fennell T Banks E Ambrogio L Cibulskis K Kernytsky A Gonzalez E Rudzicz N Engert JC DePristo MA Daly MJ Cohen JC Hobbs HH Altshuler D Schonfeld G Gabriel SB Yue P Kathiresan S Exome sequencing, ANGPTL3 mutations, and familial combined hypolipidemia. N Engl J Med 2010 363 2220 2227 10.1056/NEJMoa1002926 20942659 PMC3008575

[B14] Rosenthal EA Ronald J Rothstein J Rajagopalan R Ranchalis J Wolfbauer G Albers JJ Brunzell JD Motulsky AG Rieder MJ Nickerson DA Wijsman EM Jarvik GP Linkage and association of phospholipid transfer protein activity to LASS4. Journal of Lipid Research 2011 52 1837 1846 10.1194/jlr.P016576 21757428 PMC3173000

[B15] Sobreira NLM Cirulli ET Avramopoulos D Wohler E Oswald GL Stevens EL Ge D Shianna KV Smith JP Maia JM Gumbs CE Pevsner J Thomas G Valle D Hoover-Fong JE Goldstein DB Whole-Genome Sequencing of a Single Proband Together with Linkage Analysis Identifies a Mendelian Disease Gene. PLoS Genet 2010 6 e1000991 10.1371/journal.pgen.1000991 20577567 PMC2887469

[B16] Anastasio N Ben-Omran T Teebi A Ha KCH Lalonde E Ali R Almureikhi M Der Kaloustian VM Liu J Rosenblatt DS Majewski J Jerome-Majewska LA Mutations in SCARF2 are responsible for Van Den Ende-Gupta syndrome. Am J Hum Genet 2010 87 553 559 10.1016/j.ajhg.2010.09.005 20887961 PMC2948800

[B17] Choi M Scholl UI Ji W Liu T Tikhonova IR Zumbo P Nayir A Bakkaloglu A Ozen S Sanjad S Nelson-Williams C Farhi A Mane S Lifton RP Genetic diagnosis by whole exome capture and massively parallel DNA sequencing. Proc Natl Acad Sci USA 2009 106 19096 19101 10.1073/pnas.0910672106 19861545 PMC2768590

[B18] Götz A Tyynismaa H Euro L Ellonen P Hyötyläinen T Ojala T Hämäläinen RH Tommiska J Raivio T Oresic M Karikoski R Tammela O Simola KOJ Paetau A Tyni T Suomalainen A Exome Sequencing Identifies Mitochondrial Alanyl-tRNA Synthetase Mutations in Infantile Mitochondrial Cardiomyopathy. American journal of human genetics 2011 88 635 642 10.1016/j.ajhg.2011.04.006 21549344 PMC3146718

[B19] Becker J Semler O Gilissen C Li Y Bolz HJ Giunta C Bergmann C Rohrbach M Koerber F Zimmermann K de Vries P Wirth B Schoenau E Wollnik B Veltman JA Hoischen A Netzer C Exome Sequencing Identifies Truncating Mutations in Human SERPINF1 in Autosomal-Recessive Osteogenesis Imperfecta. American journal of human genetics 2011 88 362 371 10.1016/j.ajhg.2011.01.015 21353196 PMC3059418

[B20] Pippucci T Benelli M Magi A Martelli PL Magini P Torricelli F Casadio R Seri M Romeo G EX-HOM (EXome HOMozygosity): A Proof of Principle. Human heredity 2011 72 45 53 10.1159/000330164 21849793

[B21] Krawitz PM Schweiger MR Rödelsperger C Marcelis C Kölsch U Meisel C Stephani F Kinoshita T Murakami Y Bauer S Isau M Fischer A Dahl A Kerick M Hecht J Köhler S Jäger M Grunhagen J de Condor BJ Doelken S Brunner HG Meinecke P Passarge E Thompson MD Cole DE Horn D Roscioli T Mundlos S Robinson PN Identity-by-descent filtering of exome sequence data identifies PIGV mutations in hyperphosphatasia mental retardation syndrome. Nat Genet 2010 42 827 829 10.1038/ng.653 20802478

[B22] Rödelsperger C Krawitz P Bauer S Hecht J Bigham AW Bamshad M de Condor BJ Schweiger MR Robinson PN Identity-by-descent filtering of exome sequence data for disease-gene identification in autosomal recessive disorders. Bioinformatics 2011 27 829 836 10.1093/bioinformatics/btr022 21278187 PMC3051326

[B23] Ng SB Buckingham KJ Lee C Bigham AW Tabor HK Dent KM Huff CD Shannon PT Jabs EW Nickerson DA Shendure J Bamshad MJ Exome sequencing identifies the cause of a mendelian disorder. Nat Genet 2010 42 30 35 10.1038/ng.499 19915526 PMC2847889

[B24] Haack TB Danhauser K Haberberger B Hoser J Strecker V Boehm D Uziel G Lamantea E Invernizzi F Poulton J Rolinski B Iuso A Biskup S Schmidt T Mewes H-W Wittig I Meitinger T Zeviani M Prokisch H Exome sequencing identifies ACAD9 mutations as a cause of complex I deficiency. Nat Genet 2010 42 1131 1134 10.1038/ng.706 21057504

[B25] Ng SB Bigham AW Buckingham KJ Hannibal MC McMillin MJ Gildersleeve HI Beck AE Tabor HK Cooper GM Mefford HC Lee C Turner EH Smith JD Rieder MJ Yoshiura K-I Matsumoto N Ohta T Niikawa N Nickerson DA Bamshad MJ Shendure J Exome sequencing identifies MLL2 mutations as a cause of Kabuki syndrome. Nat Genet 2010 42 790 793 10.1038/ng.646 20711175 PMC2930028

[B26] Pierce SB Walsh T Chisholm KM Lee MK Thornton AM Fiumara A Opitz JM Levy-Lahad E Klevit RE King M-C Mutations in the DBP-deficiency protein HSD17B4 cause ovarian dysgenesis, hearing loss, and ataxia of Perrault Syndrome. Am J Hum Genet 2010 87 282 288 10.1016/j.ajhg.2010.07.007 20673864 PMC2917704

[B27] Norton N Li D Rieder MJ Siegfried JD Rampersaud E Züchner S Mangos S Gonzalez-Quintana J Wang L McGee S Reiser J Martin E Nickerson DA Hershberger RE Genome-wide Studies of Copy Number Variation and Exome Sequencing Identify Rare Variants in BAG3 as a Cause of Dilated Cardiomyopathy. American journal of human genetics 2011 88 273 282 10.1016/j.ajhg.2011.01.016 21353195 PMC3059419

[B28] Glazov EA Zankl A Donskoi M Kenna TJ Thomas GP Clark GR Duncan EL Brown MA Whole-Exome Re-Sequencing in a Family Quartet Identifies POP1 Mutations As the Cause of a Novel Skeletal Dysplasia. PLoS Genet 2011 7 e1002027 10.1371/journal.pgen.1002027 21455487 PMC3063761

[B29] Shi Y Li Y Zhang D Zhang H Li Y Lu F Liu X He F Gong B Cai L Li R Liao S Ma S Lin H Cheng J Zheng H Shan Y Chen B Hu J Jin X Zhao P Chen Y Zhang Y Lin Y Li X Fan Y Yang H Wang J Yang Z Exome Sequencing Identifies ZNF644 Mutations in High Myopia. PLoS Genet 2011 7 e1002084 10.1371/journal.pgen.1002084 21695231 PMC3111487

[B30] Le Goff C Mahaut C Wang LW Allali S Abhyankar A Jensen S Zylberberg L Collod-Beroud G Bonnet D Alanay Y Brady AF Cordier M-P Devriendt K Genevieve D Kiper PÖS Kitoh H Krakow D Lynch SA Le Merrer M Mégarbane A Mortier G Odent S Polak M Rohrbach M Sillence D Stolte-Dijkstra I Superti-Furga A Rimoin DL Topouchian V Unger S Mutations in the TGF[beta] Binding-Protein-Like Domain 5 of FBN1 Are Responsible for Acromicric and Geleophysic Dysplasias. The American Journal of Human Genetics 2011 89 7 14 10.1016/j.ajhg.2011.05.012 PMC3135800 21683322

[B31] Züchner S Dallman J Wen R Beecham G Naj A Farooq A Kohli MA Whitehead PL Hulme W Konidari I Edwards YJK Cai G Peter I Seo D Buxbaum JD Haines JL Blanton S Young J Alfonso E Vance JM Lam BL Peričak-Vance MA Whole-Exome Sequencing Links a Variant in DHDDS to Retinitis Pigmentosa. American journal of human genetics 2011 88 201 206 10.1016/j.ajhg.2011.01.001 21295283 PMC3035708

[B32] Kruglyak L Daly MJ Reeve-Daly MP Lander ES Parametric and nonparametric linkage analysis: a unified multipoint approach. American Journal of Human Genetics 1996 58 1347 1363 8651312 PMC1915045

[B33] Lander ES Botstein D Homozygosity Mapping: A Way to Map Human Recessive Traits with the DNA of Inbred Children. Science 1987 236 1567 1570 10.1126/science.2884728 2884728

[B34] The International HapMap Consortium A second generation human haplotype map of over 3.1 million SNPs. Nature 2007 449 851 861 10.1038/nature06258 17943122 PMC2689609

[B35] Bahlo M Bromhead CJ Generating linkage mapping files from Affymetrix SNP chip data. Bioinformatics 2009 25 1961 1962 10.1093/bioinformatics/btp313 19435744

[B36] Harismendy O Ng PC Strausberg RL Wang X Stockwell TB Beeson KY Schork NJ Murray SS Topol EJ Levy S Frazer KA Harismendy O Ng PC Strausberg RL Wang X Stockwell TB Beeson KY Schork NJ Murray SS Topol EJ Levy S Frazer KA Evaluation of next generation sequencing platforms for population targeted sequencing studies. Genome Biology 2009 10 R32 10.1186/gb-2009-10-3-r32 19327155 PMC2691003

[B37] Cherny SS Abecasis GR Cookson WO Sham PC Cardon LR The effect of genotype and pedigree error on linkage analysis: analysis of three asthma genome scans. Genet Epidemiol 2001 21 Suppl 1 S117 122 11793653 10.1002/gepi.2001.21.s1.s117

[B38] Li H Ruan J Durbin R Mapping short DNA sequencing reads and calling variants using mapping quality scores. Genome Research 2008 18 1851 1858 10.1101/gr.078212.108 18714091 PMC2577856

[B39] McKenna A Hanna M Banks E Sivachenko A Cibulskis K Kernytsky A Garimella K Altshuler D Gabriel S Daly M DePristo MA The Genome Analysis Toolkit: a MapReduce framework for analyzing next-generation DNA sequencing data. Genome Res 2010 20 1297 1303 10.1101/gr.107524.110 20644199 PMC2928508

[B40] Abecasis GR Wigginton JE Handling Marker-Marker Linkage Disequilibrium: Pedigree Analysis with Clustered Markers. American journal of human genetics 2005 77 754 767 10.1086/497345 16252236 PMC1271385

[B41] Browning SR Browning BL High-Resolution Detection of Identity by Descent in Unrelated Individuals. American journal of human genetics 2010 86 526 539 10.1016/j.ajhg.2010.02.021 20303063 PMC2850444

[B42] Thompson EA Inferring coancestry of genome segments in populations. Invited Proceedings of the 57th Session of the International Statistical Institute; Durban, South Africa 2009

[B43] Novoalign http://www.novocraft.com

[B44] Picard http://picard.sourceforge.net

[B45] Li H Handsaker B Wysoker A Fennell T Ruan J Homer N Marth G Abecasis G Durbin R Genome Project Data Processing S Li H Handsaker B Wysoker A Fennell T Ruan J Homer N Marth G Abecasis G Durbin R The Sequence Alignment/Map format and SAMtools. Bioinformatics 2009 25 2078 2079 10.1093/bioinformatics/btp352 19505943 PMC2723002

[B46] Linkdatagen MPS http://bioinf.wehi.edu.au/software/linkdatagen/#mps

[B47] Abecasis GR Cherny SS Cookson WO Cardon LR Merlin--rapid analysis of dense genetic maps using sparse gene flow trees.[see comment]. Nature Genetics 2002 30 97 101 10.1038/ng786 11731797

[B48] Wang K Li M Hakonarson H ANNOVAR: functional annotation of genetic variants from high-throughput sequencing data. Nucleic Acids Research 2010 38 e164 10.1093/nar/gkq603 20601685 PMC2938201

