# Supplementary material for: Reducing the exome search space for Mendelian diseases using genetic linkage analysis of exome genotypes
Source: Genome Biol. 2011 Sep 14;12(9):R85. doi: 10.1186/gb-2011-12-9-r85 (PMC3308048; doi:10.1186/gb-2011-12-9-r85)
Supplement: Additional file 1 — Supplementary tables. [file gb-2011-12-9-r85-S1.DOC]

Table S1. Alignment and post processing statistics.

|  | **M-3** | **M-4** | **A-7** | **T-1** |
| --- | --- | --- | --- | --- |
|  | **No. (%)** | **No. (%)** | **No. (%)** | **No. (%)** |
| Read Sequences | 56,642,154 (100.0) | 53,784,932 (100.0) | 61,207,166 (100.0) | 100,918,798 (100.0) |
| Aligned | 54,859,897 (96.9) | 51,882,126 (96.5) | 59,302,768 (96.9) | 97,420,051 (96.5) |
| & Unique Alignment | 50,401,923 (89.0) | 47,349,805 (88.0) | 54,051,242 (88.3) | 88,335,423 (87.5) |
| & Non-duplicate | 42,916,597 (75.8) | 40,410,291 (75.1) | 44,861,655 (73.3) | 75,332,924 (74.6) |

Table S2. Distribution of coverage across the 62,085,286 bp targeted by Illumina TruSeq capture. Only bases with base quality ≥13 from reads with mapping quality ≥13 were considered.

|  | **first quartile** | **median** | **mean** | **third quartile** | **≥5 reads (%)** | **≥10 reads (%)** |
| --- | --- | --- | --- | --- | --- | --- |
| A-7 | 17 | 42 | 46.9 | 70 | 87.6 | 82.3 |
| T-1 | 25 | 61 | 68.0 | 102 | 89.8 | 85.8 |
| M-3 | 14 | 38 | 43.5 | 66 | 86.5 | 80.3 |
| M-4 | 14 | 36 | 40.8 | 61 | 87.1 | 80.8 |

Table S3. Coverage of bases between 1 and 200 bp from a base targeted by TruSeq capture. N=74,926,531 bp. Only bases with base quality ≥13 from reads with mapping quality ≥13 were considered.

|  | **first quartile** | **median** | **mean** | **third quartile** | **≥5 reads (%)** | **≥10 reads (%)** |
| --- | --- | --- | --- | --- | --- | --- |
| A-7 | 0 | 4 | 13.8 | 19 | 37.2 | 48.1 |
| T-1 | 1 | 9 | 21.7 | 31 | 49.2 | 60.2 |
| M-3 | 0 | 5 | 13.9 | 20 | 39.8 | 52.0 |
| M-4 | 0 | 5 | 13.1 | 19 | 39.3 | 52.5 |
